# Validity and Reproducibility of a Food Frequency Questionnaire for Dietary Factors Related to Colorectal Cancer

**DOI:** 10.3390/nu9111257

**Published:** 2017-11-17

**Authors:** Daniel Nigusse Tollosa, John Van Camp, Inge Huybrechts, Lieven Huybregts, Joris Van Loco, Stefaan De Smet, Ellen Sterck, Céline Rabâi, Thomas Van Hecke, Lynn Vanhaecke, Els Vossen, Marc Peeters, Carl Lachat

**Affiliations:** 1 Department of Food Safety and Food Quality, Ghent University, Coupure Link 653, 9000 Ghent, Belgium; danielnigusse@gmail.com (D.N.T.); John.VanCamp@ugent.be (J.V.C.); ellen_sterck@hotmail.com (E.S.); celine.rabai@hotmail.com (C.R.); 2 Department of Public Health, College of Health Sciences, Mekelle University, Mekelle 1871, Ethiopia; 3 International Agency for Research on Cancer (IARC), 150 Cours Albert Thomas, 69372 Lyon CEDEX 08, France; HuybrechtsI@iarc.fr; 4 Poverty, Health and Nutrition Division, International Food Policy Research Institute, 2033 K Street NW, Washington, DC 20006, USA; L.Huybregts@cgiar.org; 5 Scientific Institute of Public Health, J. Wytsmanstraat 14, 1050 Brussels, Belgium; joris.VanLoco@wiv-isp.be; 6 Laboratory of Animal Nutrition and Animal Product Quality, Ghent University, Coupure Links 653, 9000 Ghent, Belgium; stefaan.desmet@ugent.be (S.D.S.); Thomas.VanHecke@ugent.be (T.V.H.); Els.Vossen@ugent.be (E.V.); 7 Laboratory of Chemical Analysis, Department of Veterinary Public Health and Food Safety, Ghent University, Salisburylaan 133, 9820 Merelbeke, Belgium; Lynn.Vanhaecke@ugent.be; 8 Department of Oncology, Antwerp University Hospital, Wilrijkstraat 10, 2650 Edegem, Belgium; Marc.Peeters@uza.be

**Keywords:** reproducibility, validity, FFQ’s, food diary, colorectal cancer

## Abstract

Dietary factors play a major role in the development of colorectal cancer. This study evaluated the reproducibility and validity of a 109-food item Food Frequency Questionnaire (FFQ) to measure the consumption of foods and nutrients related to the development of colorectal cancer in a population aged ≥50 years in Flanders, Belgium. A semi-quantitative FFQ was administered two times in a period of two weeks to evaluate reproducibility (FFQ1 and FFQ2). The validity of the FFQ was assessed by comparing FFQ1 against the 3-day diary method (3 DD). A total of 162 respondents (mean age 57.5 years) provided data for the FFQ, of whom 156 also participated in the validity assessment. Mean differences in the intake of foods and nutrients between FFQ1 and FFQ2 were, overall, small and statistically insignificant. However, a higher estimation was observed by FFQ1 as compared to the 3-DD method for the majority of food groups and nutrient intake in the validity assessment. A systematic mean difference (g/day) was observed for eight food groups in the Bland–Altman agreement test; the largest was for fruit intake. Regarding the nutrients, a systematic mean difference was observed in calcium, fat, and vitamin D intake. Overall, the reproducibility of the FFQ was good, and its validity could be satisfactory for estimating absolute food and nutrient intakes and ranking individuals according to high and low intake categories.

## 1. Introduction

Colorectal cancer is a major cause of cancer morbidity and mortality worldwide, and food intake plays a central role in the course of its development [1,2]. Dietary risk factors for colorectal cancer are complex. On the one hand, high consumption of meat and processed meat, but not white meat or fish, is positively correlated with the development of colorectal cancer [2,3,4,5,6]. The risk of colorectal cancer is increased by 18% per 50 g intake of processed meat, and by 17% per 100 g intake of red meat, on a daily basis [3]. In particular, heme iron in red meat and its nitrosylated form in processed meat are of concern, given their catalytic effect on the formation of *N*-nitroso-compounds and lipid peroxidation products [3,7,8]. Mutagenic compounds generated during the cooking of meat at high temperatures (heterocyclic aromatic amines and polycyclic aromatic hydrocarbons) might also interfere in this formation process [3,9]. On the other hand, other components of diet such as calcium, fruits, vegetables, and dietary fiber are considered protective against developing colorectal cancer [2,10,11,12,13].

Results from animal and in vitro studies indicate that omega-3 fatty acids, especially the long-chain polyunsaturated fatty acids eicosapentaenoic and docosahexaenoic acids, which are mainly present in fatty fish, inhibit carcinogenesis [5]. Dietary antioxidants might counteract the effect of heme iron and it was recently shown that the association between heme-iron and colorectal adenoma risk depends also on the non-enzymatic dietary antioxidant capacity [11]. This illustrates the need for assessing dietary patterns and interactions between foods when evaluating dietary risk factors in relation to chronic diseases.

Several dietary assessment methods, i.e., the 24-h dietary recall, the dietary record, and the food frequency questionnaire (FFQ), are available for epidemiological purposes. FFQs are often used in epidemiological studies to estimate long-term dietary exposure, mainly due to their applicability in large samples and their ability to categorize subjects based on their intake [14,15,16]. Measurement errors in FFQs can be estimated through relative validity studies, in which dietary intake assessed using FFQ is compared with a more precise method, such as weighted dietary records [14].

An FFQ records the intake of a limited list of carefully selected food items, and, as such, has been developed for a specific purpose and a specific dietary risk factor. In addition, as food intake and dietary habits vary by population groups, FFQs are often developed and used for specific populations. Therefore, it is important to consider the specific diet of older adults when assessing dietary risk factors of colorectal cancer accurately since more than 80% of cases of colorectal cancer are diagnosed in people aged 50 or over [1].

To date, the validity of a comprehensive FFQ to assess dietary risk factors for colorectal cancer has not been evaluated in older adults. The existing studies, albeit with some conflicting results, have been able to highlight important associations between diet and colorectal cancer using an FFQ. However, these instruments either did not provide validity estimates [17,18,19] or were validated for use in general cancer studies in which all specific dietary risk factors for colorectal cancer were not considered [20,21,22,23]. Therefore, the purpose of the present research was to evaluate the reproducibility and validity of an FFQ, which includes all dietary factors related to colorectal cancer in a population >50 years old in the Flemish region of Belgium.

## 2. Materials and Methods

The validation consisted of comparing the FFQ with the 3-day diary record for assessing the intake of foods and nutrients, while the reproducibility test consisted of comparing the food and nutrient intake calculated using the same FFQ administered within two weeks (FFQ1 and FFQ2). The 3-day diary record was distributed after the second FFQ (FFQ2) had been completed in order to avoid bias introduced by increased awareness when completing a 3-day diary.

### 2.1. Study Participants

A total number of 300 participants were contacted via oral (in person, and via telephone) and e-mail communication and invited to participate in this study conducted in the Flemish region of Belgium. Purposive sampling (specifically the snowball method) was used to select those participants through family, friends, acquaintances, colleagues, and associations. Out of this number, 176 participants replied, agreed to participate, and were enrolled in the study. Age ≥50 years and not being vegetarian were the main inclusion criteria. Data were collected from 1 March till 1 April 2015. First the FFQ was collected in a two-week interval (FFQ1 then FFQ2) followed by the diary method.

Ethical clearance was obtained from the Ethics Committee of Ghent University (number EC/2013/1124). Informed consent was obtained from the participants, and the anonymity of the participants was ensured at all times.

### 2.2. Development of the FFQ

A self-administered semi-quantitative food frequency questionnaire was developed for the purpose of the study (Supplementary material: Table S1). The FFQ included specific questions to measure the intake of food sources associated with the development of colorectal cancer. Protective and propagating foods and dietary components were identified from existing meta-analyses and systematic reviews [2,4,5,24,25,26]. Specifically, heme-iron, nitrite/nitrate, and fat intake from red and processed meat were included because these are established or putative risk factors for the development of colorectal cancer. As vitamin D, calcium, fiber, and omega-3 fatty acids are considered to be protective compounds, vegetables, dairy, fruits, fish, and whole grain products were also included. All possible foods and food groups containing these nutrients were identified from the Belgian food consumption survey conducted in 2004 [27]. For each food and nutrient associated with colorectal cancer, a regression model was fitted. The model contained the total nutrient intake as a dependent variable and the corresponding nutritional composition of the foods consumed and their consumption frequency as independent variables. Using stepwise regression analysis, food items that contributed significantly to the variability of intake of each nutrient were considered for the FFQ. A total of 109 food items were listed for inclusion in the FFQ.

The foods in this list were organized into 22 food groups (Supplementary material: Table S2) on the basis of similar nutritional properties and considering the scientific facts related to the development of colorectal cancer. For fish and meat, when appropriate, the preparation method (i.e., baked in the pan, baked in the oven, grilled, and deep-fried) and the degree of doneness (i.e., rare, medium rare, medium, medium-well, and well done) was specified, as cooking method and degree of doneness are considered important in relation to the intake of polycyclic aromatic hydrocarbons and heterocyclic amines.

The FFQ asked participants to recall the consumption frequency for the past year. Participants estimated the intake of each food with seven possible options: never or <once per month, once a month, 2–3 times per month, once a week, 2–3 times per week, 4–6 times per week, and daily consumption. The FFQ also assessed the average quantity consumed per day. Examples and photographs of different portion sizes were provided with the FFQ to assist participants when estimating the average quantity of food consumed per day.

### 2.3. 3-Day Diary

A 3-day dietary record was used as a reference method for relative validity analysis. Unlike the FFQ, the diary method is a prospective method and allows the respondent to record any food intake (quantitatively and qualitatively). For the 3-day dietary record in this study, the participants were asked to select two days during the week and one during the weekend. The days were not consecutive to increase the possibility of variety in meal consumption. For composite dishes, the participants were asked to mention the different ingredients and their quantities so that the nutrient composition could be calculated.

A detailed explanation with examples and pictures was provided to guide participants in recording their food item and portion size accurately. Despite clear instructions, however, the majority of the respondents (>50%) did not report the doneness and cooking methods of fish and meat intake. As a result, we were unable to consider the food groups related to fish and meat preparation, and meat doneness for the assessment of validity. The final number of food groups was reduced to 18 for this reason.

### 2.4. Data Management and Analysis

Data from the FFQs were extracted using TeleForm designer software 10.2 (Digital Vision, Hewlett Packard, Palo Alto, CA, USA). Errors or missing values were detected by the program and reviewed case by case. For the 3-day diary, Lucille software version 0.1 [28] was used for data entry and processing. The raw data were corrected for errors that occurred during completion and data-entering of the FFQ. In the case of absence of a response in one of the two FFQ’s, the day with recorded items was used for the validity study.

### 2.5. Calculation of Daily Food and Nutrient Intake

The average frequency of food intake per week and month of the FFQ was converted to a daily intake value (e.g., frequency of 2–3 times per month = 2.5/30.5 times per day). Values were considered missing if both the frequency and the amounts were not completed. If either the amount or the frequency only was reported, we first considered the trend in a similar food group that would estimate the amount of intake or the frequency. If this was not possible, we took the median value from distribution of that particular variable, i.e., food or nutrient intake. However, this happened for only a few variables. Once the average daily intake of each food type was calculated, the nutrient intake per day was computed using the following formula: nutrient intake per day = ∑ (daily intake of food (consumption frequency) × amount of nutrient in the food × amount of consumed food).

For the 3-day diary, to account for non-uniform intake of some nutrients throughout the week, e.g., alcohol, a weighting factor was assigned to compensate for the difference in dietary intake between weekdays and weekend days. To calculate the average intake per day, the weighting factor was multiplied by the amount consumed per foodstuff, i.e., average daily intake = ((weekend intake × 2) + (average weekday intake × 5))/7.

The nutritional composition of foods consumed (calcium, vitamin D, fiber, omega-3 fatty acids (protective association), and fat, alcohol, heme-iron and nitrate/nitrites (propagative association)) was obtained from the Belgian [29] or the Dutch [30] food composition table. In the absence of specific data in the latter databases, a heme-iron database was created from the values reported by Cross et al. [31]. Nitrate/nitrite was obtained from specific analysis of food from the Belgian Scientific Institute of Public Health. An overview of the data on nitrate and nitrite in various foods (including different types of vegetables, fruits, potatoes, meat, and dairy products) as used in present study has been described previously [32].

### 2.6. Data Analysis

Estimates of dietary intake from the FFQs and 3-day diary, as well as the differences estimated by the two methods, were verified for normality. As both distributions deviated significantly from a normal distribution, in addition to the mean value, median and inter-quartile range (IQR) were used to describe the estimates.

Measurement agreement for the reproducibility and validity was assessed using the Bland-Altman plots, whereby the difference of food and nutrient intake between methods was plotted against the average intake estimated by both methods [33]. In case mean differences were associated with the estimated intake level, data were log-transformed prior to assessing measurement agreement. Although the use of correlation tests to assess measurement agreement has limitations [33,34], Spearman correlation statistics are provided to enable comparison with previous studies.

Cohen’s kappa statistic was used to assess the agreement of classifying participants into similar quartiles of daily intake. To enable comparison with other validity studies, we applied a Wilcoxon signed rank test and Spearman correlation to compare both methods. STATA (version 13, Stata Corp LP, College Station, TX, USA) was used to analyze the data.

## 3. Results

### 3.1. Study Participant Characteristics

A complete response for all measurements was obtained from 162 participants for both times the FFQs were administered. Due to incompleteness of the data in the diaries, we were unable to consider one male and five female participants in the validation analysis. Hence, a total of 156 (96.8%) participants provided data for the validity analysis. The mean age of the participants was 57.7 years (Standard Deviation = 7.2 years), and the majority of the participants were female with a higher degree of education (Table 1). Mean energy intake as measured from the diary was 2127.7 ± 1319.3 kcal.

### 3.2. Reproducibility Analysis

Compared with the FFQ2, a slightly higher estimated intake was observed in the FFQ1 for the majority of foods (18/22 food groups) and nutrients (7/8 nutrients) (Table 2). The mean difference (MD) between FFQ1 and FFQ2 (FFQ1–FFQ2) was statistically significant for total meat (MD = 10.2, 95% Confidence Interval (CI) = 1.7, 18.6), total red meat (MD = 7.4, 95% CI = 0.8, 13.9), total red meat and processed poultry (MD = 7.3, 95% CI = 0.2, 15.3), total fresh meat (MD = 5.7, 95% CI = 0.3, 10.9) and whole wheat grain products (MD = 12.3, 95% CI = 2.7, 21.2) (Table 3). This difference, however, was a maximum of 12 g for those food groups that had a significant mean difference between the first and second administration. The correlation between FFQ1 and FFQ2 was high for all foods, ranging from *ρ* = 0.74 for total poultry and fresh poultry to *ρ* = 0.91 for milk. Good classification agreement of estimates by FFQ1 and FFQ2 was also observed (60–80%). The large limits of agreement, in particular for alcohol, whole wheat grain, dairy products, vegetables, fruit, and milk, however, indicate large differences in estimated intake at the individual level.

Total intake of heme-iron was estimated to be significantly higher in FFQ1 compared to FFQ2 (MD = 0.3, 95% CI = 0.03, 0.4). The mean differences in absolute nutrient intake between FFQ1 and FFQ2, however, were all less than 5%. A mean difference, albeit small (0.1 mg/day), between the FFQ1 and FFQ2 estimates was observed for omega-3 and nitrate (Table 3). High correlation coefficients were found for all nutrients; these ranged from *ρ* = 0.77 for vitamin D intake to *ρ* = 0.94 for alcohol intake. The kappa value (*k*) ranged from *k* = 0.47 for the total vitamin D intake to *k* = 0.77 for the total alcohol intake, indicating a moderate to very good classification agreement. The individual intake differences between FFQ1 and FFQ2 varied widely in particular for calcium (MD = 71.4 mg/day) and fat intake (MD = 6.9 g/day).

### 3.3. Relative Validity Analysis

For the majority (>75%) of the 18 food groups, the FFQ1 estimated a higher intake compared to the 3-day diary (Table 4). The correlation between the estimated intakes of the FFQ1 compared to the diary ranged from *ρ* = 0.14 to *ρ* = 0.74, indicating a correlation ranking from very weak for fresh meat to strong for alcohol intake. For the majority (11/18) of food groups, the Spearman correlation was low (<0.35). The highest correct classification into the same quartile was observed for “alcohol” and “dairy products”—i.e., 52.5% and 47.4%, respectively. For the other food groups, the classification agreement ranged from 30.1% for red meat to 39.8% for total fish (fresh and processed fish) (Table 4).

A systematic mean difference (g/day) was observed for almost half of the food groups (8/18), and mainly for meat (red meat and poultry) products. The largest significant mean difference was observed for fruits (65.2 g/day), followed by total fresh meat (36 g/day).

Except for daily fiber intake, the FFQ1 estimated a higher nutrient intake than the dietary record method. The highest difference was observed in calcium, fat, and vitamin D intake. The median difference also ranged from 0.5 mg/day for nitrate intake to 245.1 mg/day for calcium. Except for fiber (MD = 0.1, 95% CI = −2.7, 2.9) and alcohol intake (MD = −1.7, 95% CI = −4.8, 1.4), a significant difference was observed between FFQ1 and the 3-day diary in other nutrients.

**Figure 1:**
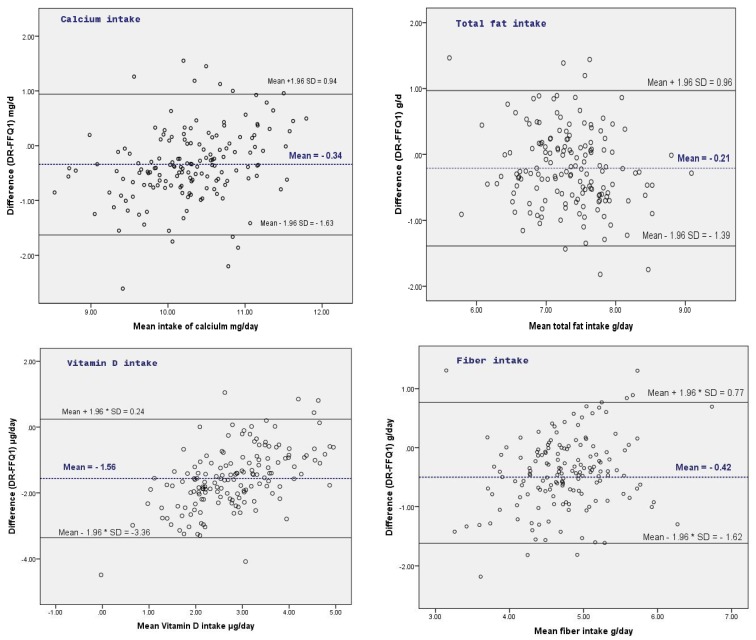

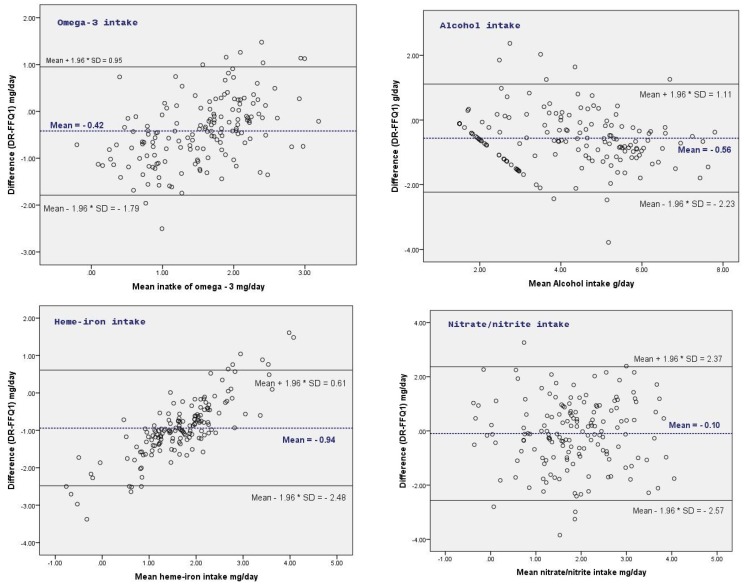
Bland and Altman plots for the log-transformed data of nutrients between the 3-days diary (DR) and FFQ1 (*n* = 156).

A high correlation between both methods was only observed for total alcohol intake (*ρ* = 0.71). For fat, omega-3, and nitrate/nitrite intake, however, the correlation of quantities estimated was very low. Low classification agreement was also observed for all nutrients except for alcohol intake (Table 5). Since differences in nutrient intakes were associated with the mean measurement, data related to the nutrient intake were log transformed for Bland and Altman statistics. The result showed that a systematic mean difference between FFQ1 and 3-day diary was observed for all the nutrients except for alcohol and fiber intake. However, the 95% limits of agreement were rather small (Figure 1).

## 4. Discussion

The aim of this study was to assess the reproducibility of the FFQ and assess relative validity against a 3-day diary method in a population of older adults in Flanders. The overall dietary intake was considered in the present study by assessing the intake of 109 food items related to colorectal cancer in a FFQ.

In terms of reproducibility, the estimated intake of foods and nutrients by FFQ1 was comparable to FFQ2. Although a few systematic differences were observed, these were small overall compared to the average daily intake. Good reproducibility was also observed from the correlations and classification agreement tests. Reproducibility in the present study was higher compared to most other studies investigating reproducibility of FFQs where correlation coefficients typically range between 0.5 and 0.7 [14]. However, these high correlations may be explained by the design and study population. First, the period between administering the FFQs was relatively short, resulting in higher reproducibility [14]. Second, the reproducibility of an FFQ in older adults is commonly higher compared to other populations, as dietary habits are more established in this population group and hence easier to recall [35,36].

In the present study, the correlation coefficients for food and nutrient intakes derived from FFQ1 and FFQ2 ranged from 0.74–0.94, which is higher than the studies by Forster et al. [37] and Jackson et al. [15], who reported coefficients ranging from 0.65–0.90 and 0.42–0.71 for food and nutrient intake, respectively. Measurement differences from individual respondents, however, varied widely and indicate that satisfactory results for reproducibility applied particularly at population group level.

Compared to the 3-day diary, however, the estimated mean intake of various foods (i.e., meat products and vegetables) and nutrients (i.e., calcium, fat, and vitamin D), as measured by the FFQ, were substantially higher. Previous studies have reported that FFQs usually overestimate the food and nutrient values of calcium and vitamins compared to other dietary assessment methods [15,38,39].

Except for vegetables, alcoholic drinks, processed red meat, and total processed meat intake, other food groups were overestimated by the FFQ compared to the diary. This overestimation might be mainly related to some food items in the FFQ that may not have been consumed during the three days of recall, and also the differences in terms of data collection, questionnaire structure, and time between the surveys. Although the difference was not statistically significant, large mean difference between the FFQ and the diary was observed for alcohol intake (135.4 g/day). This is in contrast to a previous validation study in Flanders that found similar estimates for alcohol intake on a daily basis from an FFQ compared to a dietary record and high correlation coefficient (*ρ* = 0.94) between both methods [40]. Validity estimates of foods that are frequently consumed have been reported as higher than foods that are periodically consumed [41].

A validation study of an FFQ in older adults in the Netherlands reported similar correlation coefficients (*r* = 0.78) as observed in the present study, and lower estimated alcohol intake as assessed by the FFQ compared to the 24 h recall on a daily basis (196 g vs. 217 g, respectively) [42]. We hypothesize that the FFQ estimations of usual alcohol intake are an underestimation of the actual consumption [43].

A low correlation coefficient was found between the FFQ and 3-day diary for the majority of food groups (<0.30). Only alcohol (0.71) and fiber (0.44) intake showed a strong and moderate correlation, respectively. The lowest classification agreement was observed for omega-3 fatty acids and nitrite/nitrate, and the highest for alcohol intake. The present findings are comparable to other studies conducted among adults or older age groups. Previous FFQ validation studies reported a correlation coefficient ≥0.3 [44,45] and a kappa ≥0.4 [45,46] for the majority of food groups considered.

The wide limits of agreement for the estimated food group and nutrient intakes between FFQ and 3-day diary indicated that the measurement agreement was inadequate at the individual level. Inconsistency was also observed across the intake level of food groups and nutrients whereby the mean differences increased as the intake level further increased, indicating the agreement between FFQ and 3-day diary was better at lower, rather than higher, average intake values. As such, the FFQ can be considered appropriate to estimate the absolute intakes at group level. After log transformation, however, the limit of agreement for nutrients was much improved (quite small), indicating the satisfactory measurement agreement at individual level.

Strengths of the present study are the use of multiple statistical methods used to assess the validity and reproducibility of the FFQ and the use of 3 non-consecutive days of dietary recording. The sample size of the present study corresponds to median sample size (*n* = 255) of FFQ validation studies [14]. However, there are some limitations that need to be considered when interpreting the results. First, the FFQ contained 109 food items, which imposed a considerable burden on the participants. The low number of questionnaires that had to be discarded due to insufficient data indicates satisfactory compliance of the participants. Although this food list was substantial, it is only slightly higher than the median number of 88 food items in FFQs [14]. Second, total energy intake was not assessed by the FFQ and could not be used to adjust validity estimates. Third, both the FFQ and diary were self-reported. Although care was taken to provide clear instructions on how to fill out the form, some misreporting cannot be ruled out. It should also be noted that the results obtained and the conclusions drawn from this study are limited to the older age groups (≥50 years). In addition, seasonal effects were not assessed. It should also be noted that the 3-day diary has limitations, particularly for estimating usual intakes of foods not consumed on a daily or regular basis such as fish intake and the intake of other specific food groups. Therefore, the relative validity results should be interpreted with caution as low correlations and large differences may also be due to measurement errors in the 3-day diaries for estimating specific foods and food groups. Finally, the FFQ was developed and validated for dietary risk factors of colorectal cancer. As risk factors for other cancers differ, the validity of the FFQ for other types of cancers or tumors remains to be investigated.

## 5. Conclusions

The FFQ showed good reproducibility for more than 85% of the food groups and nutrients in this study. Overall relative validity is satisfactory and could be suitable for estimating absolute food and nutrient intakes, and for ranking individuals in high and low intake categories. There was a better agreement between the FFQ and 3-day diary at the lower nutrient and food intake level. Estimates of omega-3 fatty acids and nitrate/nitrite, however, should be interpreted with caution.

## Figures and Tables

**Table 1:** Demographic characteristics of the sample, *n* = 162 for reproducibility and 156 for validity.

Characteristics	*n* (%)	
	Reproducibility	Validity
Sex		
Male	66 (40.7)	65 (41.7)
Female	96 (59.3)	91 (58.3)
Education		
Primary education	5 (3.1)	4 (2.6)
Secondary education	67 (42.2)	64 (42.1)
Higher education	87 (54.7)	84 (55.3)
Occupation		
Unemployed	7 (4.3)	7 (4.5)
Blue collar worker	10 (6.2)	10 (6.4)
Clerk	67 (41.4)	62 (39.7)
Executive and manager	14 (8.6)	14 (9.0)
Private work	19 (11.7)	19 (12.2)
Retired	45 (27.8)	44 (28.2)
Age group		
50–55	76 (46.9)	75 (48.1)
>55 to ≤65	51 (31.5)	49 (31.4)
>65 to ≤75	30 (18.5)	28 (17.9)
>75	5 (3.1)	4 (2.6)

**Table 2:** Descriptive statistics (mean, median, and IQR) for reproducibility (*n* = 162) and validity (*n* = 156) analysis.

	Mean			Median			IQR (25%, 75%)		
Foods and nutrients	FFQ1	FFQ2	Diary	FFQ1	FFQ2	Diary	FFQ1	FFQ2	Diary
Food									
Fish									
Total fish (fresh and processed fish) (g)	45.7	44.5	36.5	36.3	36.1	33.3	22.3, 53.4	23.2, 51.9	<0.01, 58.3
Fresh fish (g)	35.1	34.2	29.8	31.3	30.9	<0.01	17.8, 43.0	20.0, 40.9	<0.01, 50.0
Processed fish (g)	8.6	9.4	6.7	3.1	4.0	<0.01	<0.01, 7.6	<0.01, 8.4	<0.01, <0.01
Fresh fish intake by cooking method (g)	33.9	32.7	-	30.9	28.9	-	16.5, 41.1	16.5, 40.1	-
Total meat									
Total meat (fresh and processed red meat, and fresh and processed poultry meat) (g)	167.6	157.4	132.8	152.5	148.6	110.0	106.3, 206.1	105.3, 198.0	79.2, 166.7
Total fresh meat (fresh red and poultry meat) (g)	111.1	105.4	75.1	103.7	101.7	59.1	74.2, 137.4	68.7, 138.3	33.3, 104.2
Total processed meat (processed red meat and poultry meat) (g)	54.5	50.1	57.7	43.7	40.2	39.2	21.4, 72.1	20.7, 66.7	16.7, 79.2
Total red meat and processed poultry meat (g)	127.8	120.5	111.7	110.9	107.4	96.7	75.6, 157.7	70.9, 154.6	57.5, 145.0
Red meat									
Total red meat (fresh and processed red meat) (g)	119.5	112.1	104.9	103.5	99.9	94.2	67.9, 147.9	65.7, 147.9	50.7, 137.0
Fresh red meat (g)	73.7	70.3	53.9	62.3	61.5	50.0	38.0, 97.6	37.1, 95.7	<0.01, 86.6
Processed red meat (g)	45.4	41.7	50.9	38.0	32.9	33.3	15.0, 58.6	15.1, 53.2	15.0, 66.7
Red meat intake by cooking method (g)	72.9	68.9	-	62.0	59.9	-	38.0, 97.0	35.4, 92.4	-
Red meat intake by doneness level (g)	72.6	69.5	-	62.3	60.7	-	37.3, 97.0	35.4, 95.7	-
Poultry meat									
Total poultry (fresh and processed poultry meat) (g)	46.9	43.4	27.9	38.0	31.9	<0.01	22.8, 64.4	21.4, 63.3	<0.01, 40.8
Fresh poultry meat (g)	37.8	35.0	21.2	28.6	25.9	<0.01	21.4, 53.6	21.0, 48.3	<0.01, 33.3
Processed poultry meat (g)	9.1	8.4	6.7	4.6	4.8	<0.01	<0.01, 12.8	0.0, 11.4	<0.01, <0.01
Alcoholic drink (g)	192.6	190.0	327.0	99.2	106.7	141.7	24.8, 244.3	28.0, 260.9	<0.0, 296.6
Whole wheat grain products (g)	175.9	163.6	157.5	167.1	151.6	137.8	117.8, 209.5	110.8, 196.8	103.8, 190.0
Dairy products (incl. milk) (g)	275.7	280.2	248.4	201.8	190.0	200.0	102.5, 329.9	94.8, 342,6	97.8, 346.7
Vegetables (g)	321.7	315.5	414.3	279.9	251.5	297.5	161.4, 399.8	152.6, 413.3	153.3, 546.3
Fruits excl. juices (g)	270.9	273.2	205.7	216.4	204.8	172.5	101.2, 326.4	116.1, 383.2	66.7, 306.7
Milk (g)	103.9	107.5	-	99.2	111.6	-	<0.01, 150.0	<0.01, 146.4	-
Nutrients									
Calcium (mg)	1236.3	1164.9	945.2	1046.6	939.8	801.5	669.3, 1505.2	618.9, 1434.4	567.5, 1169.0
Total fat (g)	167.4	160.5	111.3	138.4	136.9	96.9	98.6, 197.7	94.12, 172.9	61.2, 189.2
Vitamin D (μg)	16.4	15.9	5.4	12.4	11.7	3.7	8.2, 20.9	8.1, 20.3	2.2, 6.9
Fiber (g)	20.8	20.1	24.4	17.9	16.7	20.7	13.8, 24.8	13.4, 24.1	15.2, 28.5
Omega-3 fatty acids (mg)	3.5	3.4	2.3	2.9	2.7	2.0	1.8, 3.9	1.8, 3.9	0.9, 3.3
Alcohol (g)	30.1	30.2	28.4	15.6	16.6	10.8	4.2, 43.2	4.9, 46.5	<0.01, 23.4
Heme iron (mg)	4.1	3.8	2.8	3.7	3.6	2.0	2.7, 4.9	2.6, 4.8	1.2, 3.0
Nitrate/nitrite (mg)	6.4	6.2	4.0	3.8	3.2	3.3	2.1, 6.6	2.0, 6.5	1.5, 4.7

All estimates are averages per day; FFQ: Food Frequency Questionnaire, IQR: Inter Quartile Range; Note that the word “meat” includes red meat and poultry. Supplementary Materials: Table S2 provides a short description of the food groups; (-) No valid estimates were obtained from the diary registrations.

**Table 3:** Reproducibility of food and nutrient intake between FFQ1 and FFQ2, *n* = 162.

Foods and nutrients	Spearman Correlation (rho)	Kappa Test		Wilcoxon Signed Rank Test—*p* Value	Bland and Altman Statistics	
		% of Correct Classification	Kappa Value		Mean Difference (95% CI)	95% Limit of Agreement
**Food**						
Fish						
Total fish (fresh and processed fish) (g)	0.82 **	63.3	0.51	0.62	1.2 (−3.1, 4.9)	−50.2, 52.0
Fresh fish (g)	0.85 **	67.7	0.57	0.31	0.9 (−1.4, 3.2)	−28.3, 30.1
Processed fish (g)	0.82 **	67.5	0.56	0.12	−0.8 (−3.5, 2.0)	−35.6, 34.1
Fresh fish intake by cooking method (g)	0.77 **	63.9	0.51	0.10	1.2 (−1.4, 3.7)	−31.2, 33.5
Total red meat and poultry						
Total meat (fresh and processed red meat, and fresh and processed poultry meat) (g)	0.80 **	59.6	0.46	0.06	10.2 (1.7, 18.6) *	−98.2, 118.5
Total fresh meat (fresh red meat and poultry meat) (g)	0.78 **	67.1	0.56	0.18	5.7 (0.3, 10.9) *	−62.4, 73.6
Total processed meat (processed red meat and poultry meat) (g)	0.77 **	62.1	0.49	0.40	4.4 (−0.9, 9.6)	−63.1, 71.9
Total red meat and processed poultry meat (g)	0.82 **	62.7	0.50	0.37	7.3 (0.2, 15.3) *	−88.8, 104.2
Red meat						
Total red meat (fresh and processed red meat) (g)	0.83 **	60.2	0.47	0.14	7.4 (0.8, 13.9) *	−76.6, 91.3
Fresh red meat (g)	0.81 **	65.5	0.54	0.32	3.4 (−1.0, 7.7)	−52.6, 59.3
Processed red meat (g)	0.79 **	60.8	0.47	0.09	3.7 (−0.4, 8.2)	−51.2, 58.9
Red meat intake by cooking method (g)	0.79 **	64.6	0.52	0.16	4.0 (−0.6, 8.5)	−54.2, 62.1
Red meat intake by doneness level (g)	0.81 **	65.2	0.53	0.26	3.1 (−0.9, 7.9)	−52.8, 59.8
Poultry meat						
Total poultry (fresh and processed poultry meat) (g)	0.74 **	61.4	0.48	0.08	3.5 (−0.9, 6.38)	−44.0, 49.5
Fresh poultry meat (g)	0.74 **	67.1	0.56	0.09	2.8 (−0.4, 5.0)	−32.4, 36.9
Processed poultry meat (g)	0.75 **	73.3	0.64	0.96	0.7 (−1.5, 2.5)	−24.9, 25.8
Alcoholic drinks (g)	0.94 **	77.1	0.69	0.87	2.6 (−13.6, 18.8)	−205.1, 210.4
Whole wheat grain products (g)	0.76 **	64.6	0.52	0.005	12.3 (2.7, 21.2) *	−106.7, 130.6
Dairy products incl. milk (g)	0.86 **	72.1	0.62	0.19	−4.5 (−36.2, 27.1)	−409.9, 400.7
Vegetables (g)	0.82 **	63.9	0.51	0.78	6.2 (−14.2, 25.9)	−249.8, 261.5
Fruits excl. juice (g)	0.87 **	71.4	0.61	0.29	−2.3 (−20.9, 6.9)	−243.8, 239.8
Milk (g)	0.91 **	81.3	0.72	0.23	−3.6 (−15.2, 7.4)	−149.0, 141.2
**Nutrients**						
Calcium (mg)	0.79 **	70.1	0.60	0.13	71.4 (−13.8, 156.5)	−1003.1, 1165.7
Total fat (g)	0.83 **	77.8	0.66	0.07	6.9 (−2.7, 16.4)	−115.9, 129.7
Vitamin D (μg)	0.77 **	60.5	0.47	0.03	0.5 (−0.6, 1.6)	−13.54, 14.51
Fiber (g)	0.82 **	66.0	0.54	0.15	0.7 (−0.3, 1.7)	−12.3, 13.8
Omega-3 fatty acids (mg)	0.83 **	67.5	0.56	0.08	0.1 (−0.1, 0.3)	−2.4, 2.6
Alcohol (g)	0.94 **	82.9	0.77	0.92	−0.1 (−3.1, 3.0)	−39.3, 39.1
Heme iron (mg)	0.81 **	65.2	0.53	0.05	0.3 (0.03, 0.4) *	−2.1, 2.5
Nitrate/nitrite (mg)	0.89 **	70.8	0.59	0.93	0.1 (−0.4, 0.6)	−6.4, 6.6

All estimates are averages per day; * *p*-Value< 0.05 ** *p*-Value < 0.01.

**Table 4:** Relative validity of food and nutrient intake when comparing the FFQ to the 3-day dietary record (*n* = 156).

Food Group	Spearman Correlation (Rho)	Kappa Test		Wilcoxon Signed Rank Test (*p* Value)	Bland and Altman Statistics	
		% Correct Classification	Kappa Value		Mean Difference (3 DD-FFQ1) (95% CI)	95% Limits of Agreement
Fish						
Total fish (fresh and processed fish) (g)	0.39 **	39.8	0.19	<0.001	9.2 (−1.2, 17.1)	−106.2, 122.2
Fresh fish (g)	0.35 *	33.3	0.11	<0.001	5.4 (−1.8, 13.1)	−88.4, 99.7
Processed fish (g)	0.16 **	33.7	0.14	<0.001	2.0 (−2.5, 6.9)	−56.7, 61.1
Total red meat and poultry meat						
Total meat (fresh and processed red meat, and fresh and processed poultry meat) (g)	0.23 *	31.4	0.08	<0.001	34.8 (17.3, 51.9) *	−185.4, 254.5
Total fresh meat (fresh red and poultry meat) (g)	0.14 *	32.1	0.09	<0.001	36 (22.5, 48.3) *	−127.9, 198.7
Total processed meat (processed red meat and poultry meat) (g)	0.38 **	35.9	0.14	0.46	−3.2 (−13.6, 7.9)	−138.4, 132.8
Total red meat and processed poultry meat (g)	0.31 **	34.6	0.12	0.03	16.1 (1.6, 30.7) *	−167.7, 200.1
Red meat						
Total red meat (fresh and processed red meat) (g)	0.28 **	30.1	0.07	0.03	14.6 (0.7, 28.2) *	−158.4, 187.3
Fresh red meat (g)	0.23 *	34.6	0.12	<0.001	19.8 (9.8, 28.3) *	−98.0, 136.0
Processed red meat (g)	0.36 **	33.9	0.12	0.919	−5.5 (−14.8, 4.4)	−126.5, 116.1
Poultry meat						
Total poultry (fresh and processed poultry meat) (g)	0.26 **	31.8	0.09	<0.001	19 (9.9, 28.2) *	−101.1, 138.6
Fresh poultry meat (g)	0.26 **	30.7	0.08	<0.001	16.6 (8.0, 24.8) *	−90.3, 123.0
Processed poultry meat (g)	0.33 **	33.3	0.06	<0.001	2.4(−1.3, 6.1)	−44.1, 48.9
Alcoholic drinks (g)	0.74 **	52.5	0.36	0.28	−135.4 (−400.1, 29.2)	−3500.0, 3211.6
Whole wheat grain products (g)	0.44 **	37.8	0.17	<0.001	18.4 (−2.0, 39.5)	−242.8, 280.4
Dairy products incl. milk (g)	0.45 **	47.4	0.29	0.22	27.3 (−14.3, 81.6)	−572.4, 639.6
Vegetables (g)	0.31 **	35.2	0.13	0.02	−92.6 (−161.3, 3.5)	−960.8, 775.9
Fruits excl. juice (g)	0.43 **	39.7	0.19	<0.01	65.2 (27.2, 107.7) *	−441.6, 576.5

All estimates are averages per day; * *p*-Value less than 0.05; ** *p*-Value less than 0.01.

**Table 5:** Validity of nutrient intake: Spearman correlation, Kappa test, Wilcoxon signed rank test, and Bland-Altman statistics (*n* = 156).

Nutrients	Spearman Correlation (rho)	Kappa Test		Wilcoxon Signed Rank Test (*p* Value)	Bland–Altman Statistics		Bland–Altman Statistics for the Log-Transformed Data	
		% Correct Classification	Kappa Value		Mean Difference (95% CI)	95% Limit of Agreement	Mean Difference (95% CI)	95% Limits of Agreement
Calcium (mg)	0.26 **	38.5	0.17	<0.001	−291.1 (−363.9, −219.2) *	−2300.0, 1459.5	−0.3 (−0.5, −0.2) *	−1.7, 1.0
Total fat (g)	0.19 *	31.4	0.08	<0.001	−41.1 (−56.1, −26.3) *	−229.4, 147.0	−0.2 (−0.3, −0.1) *	−1.4, 1.0
Vitamin D (µg)	0.22 **	34.6	0.12	<0.001	−14.3 (−16.6, −11.9) *	−44.1, 15.6	−1.6 (−1.7, −1.4) *	−3.4, 0.3
Fiber (g)	0.44 **	34.6	0.12	0.397	0.1 (−2.7, 2.9)	−35.7, 35.8	−0.4 (−0.5, 0.3) *	−1.6, 0.8
Omega 3 fatty acids (mg)	0.15	29.5	0.05	<0.001	−1.3 (−1.7, −0.89) *	−6.5, 3.9	−0.4 (−0.5, −0.3) *	−1.8, 1.0
Alcohol (g)	0.71 **	50.0	0.33	0.554	−1.7 (−4.8, 1.4)	−41.8, 42.3	0.1(−0.04, 0.3)	−1.6, 1.8
Heme iron (mg)	0.23 **	36.5	0.15	<0.001	−1.2 (−1.8, −0.7) *	−8.7, 6.2	−0.9 (−1.1, −0.8) *	−2.5, 0.6
Nitrate/nitrite (mg)	0.06	30.5	0.07	<0.001	−2.5 (−3.9, −1.0) *	−20.7, 15.8	−0.1(−0.3, −0.1) *	−2.6, 2.4

CI: Confidence Interval; * *p*-Value < 0.05; ** *p*-Value < 0.01.

## Supplementary Materials

The following are available online at www.mdpi.com/2072-6643/9/11/1257/s1, Table S1: Food Frequency Questionnaire, Table S2: Food group list of the food frequency questionnaire and short description.

## Abbreviations

CI	Confidence Interval
FFQ	Food Frequency Questionnaire
IQR	Inter Quartile Range
MD	Mean Difference
